# Mesenchymal stem cell transformation and sarcoma genesis

**DOI:** 10.1186/2045-3329-3-10

**Published:** 2013-07-23

**Authors:** Wei Xiao, Alexander B Mohseny, Pancras C W Hogendoorn, Anne-Marie Cleton-Jansen

**Affiliations:** 1 Department of Pathology, Leiden University Medical Center, Albinusdreef 2, Leiden, 2333ZA, the Netherlands

**Keywords:** MSC, Sarcoma, Bone tumour, Soft tissue tumour, Osteosarcoma, Ewing sarcoma

## Abstract

MSCs are hypothesized to potentially give rise to sarcomas after transformation and therefore serve as a good model to study sarcomagenesis. Both spontaneous and induced transformation of MSCs have been reported, however, spontaneous transformation has only been convincingly shown in mouse MSCs while induced transformation has been demonstrated in both mouse and human MSCs. Transformed MSCs of both species can give rise to pleomorphic sarcomas after transplantation into mice, indicating the potential MSC origin of so-called non-translocation induced sarcomas. Comparison of expression profiles and differentiation capacities between MSCs and sarcoma cells further supports this. Deregulation of P53- Retinoblastoma-, PI3K-AKT-and MAPK pathways has been implicated in transformation of MSCs. MSCs have also been indicated as cell of origin in several types of chromosomal translocation associated sarcomas. In mouse models the generated sarcoma type depends on amongst others the tissue origin of the MSCs, the targeted pathways and genes and the differentiation commitment status of MSCs. While some insights are glowing, it is clear that more studies are needed to thoroughly understand the molecular mechanism of sarcomagenesis from MSCs and mechanisms determining the sarcoma type, which will potentially give directions for targeted therapies.

## Introduction

**Figure 1:**
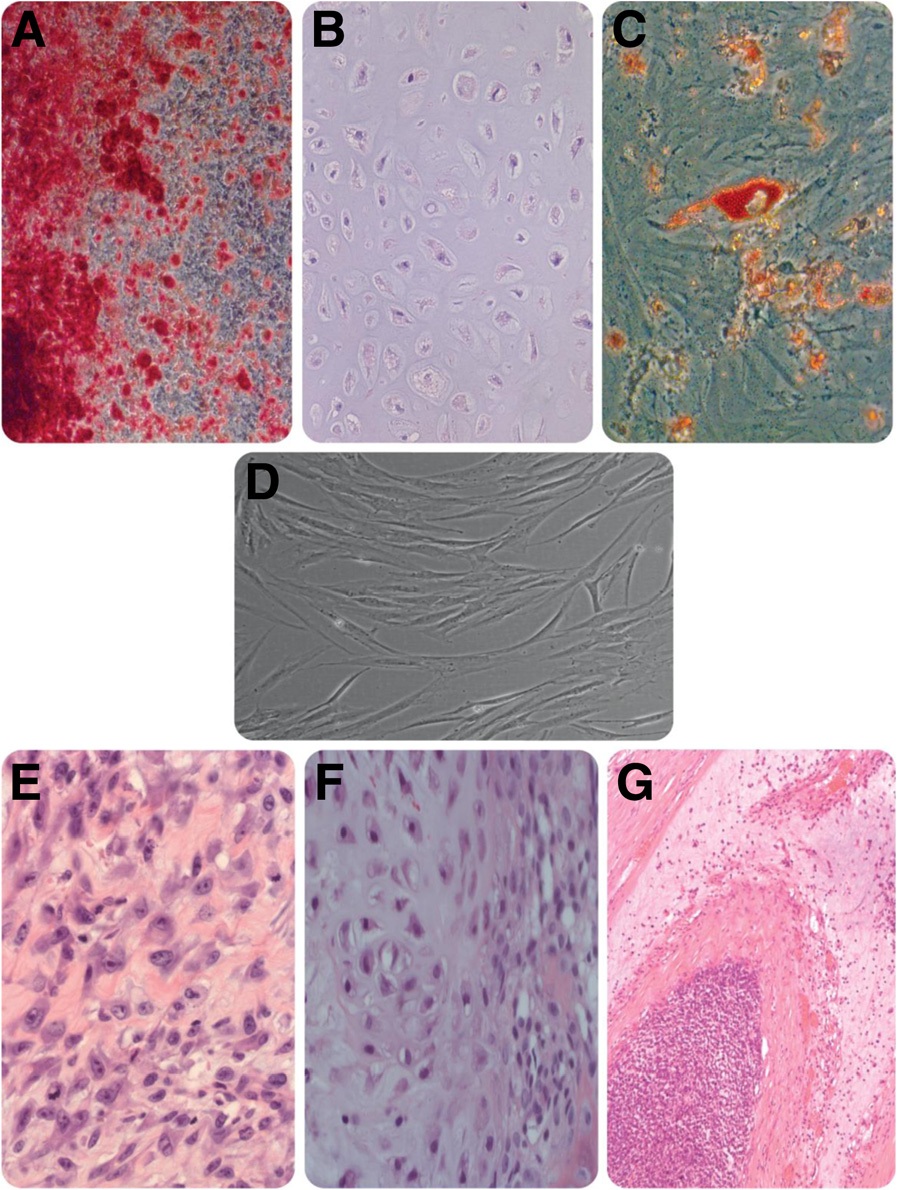
**Trilineage differentiation capacity of human MSCs and representative types of sarcomas.** Human MSCs are capable of differentiating into osteoblasts, chondrocytes and adipocytes under proper inductions. This differentiation spectrum corresponds with the histological spectrum of different types of sarcomas, represented here by osteosarcoma, chondrosarcoma and liposarcoma. This correlation supports the hypothesis that MSCs are the cell of origin of sarcomas. **A**: alizarin red staining for osteoblast differentiation assay, **B**: toluidine blue staining for chondrocyte differentiation assay of MSC pellets, **C**: oil red staining for adipocyte differentiation. **D**: human MSC cell culture. **E**: osteosarcoma, **F**: chondrosarcoma, **G**: liposarcoma.

MSCs have been under intensive research and application efforts since their first establishment by Friedenstein and his colleagues in 1968 [1]. Standard criteria developed by the International Society for Cellular Therapy define MSCs by three characteristics: 1) plastic adherence under standard culture conditions, 2) expression of CD105, CD73 and CD90 and no expression of CD45, CD34, CD14, CD11b, CD79b, CD19 and HLA-DR and 3) capacity to differentiate into osteoblasts, chondroblasts and adipocytes *in vitro*, termed trilineage differentiation potential (Figure 1) [2].

Owing to the ease of isolation, expansion, the multi-lineage differentiation potential and a variety of physiological functions, MSCs are applied in a wide range of experimental and medical applications. Among them are the enhancement of hematopoietic stem cell engraftment, the amelioration of acute graft-versus-host disease, cardiac diseases and regenerative medicine approaches for especially bone and cartilage [2].

Cell transformation is a process during which genetic changes occur, resulting in cells with the ability to grow indefinitely and anchorage-independently and with tumorigenic properties upon transplantation [3-7]. Senescence has been overcome in these transformed cells [4,8-10]. On one hand, the potentials of MSCs to transform, to initiate sarcomas and under some conditions to facilitate tumour progression are calling for caution for MSC-based applications [5,11]. On the other hand, the transforming property of MSCs and their possible role as sarcoma progenitors make these cells useful for studying sarcomagenesis and progression. In this review we present an overview of the roles of MSCs in sarcomas, with a specific focus on tumorigenic transformation and sarcomagenesis.

## MSC transformation

### Spontaneous mouse MSC transformation

Mouse MSCs have been consistently demonstrated to spontaneously undergo tumorigenic transformation after long term *ex vivo* culture [4,6]. This transformation process can also be induced by certain manipulations, including both gene targeting and drug or chemical treatment to affect crucial pathways (Table 1) [11-14]. In contrast, human MSCs do not spontaneously transform *in vitro*, even after long term culturing, which will be discussed later in more detail [9,13,15].

**Table 1:** A summary of spontaneous transformation studies with mouse BMMSCs

**Transformation after**	**Type of sarcoma**	**Associated genetic event(s)**	**Reference**
Long term culture	Osteosarcoma	Aneuploidy + CDKN2A/p16 loss	[4]
		Abnormal karyotype	[6]
	Fibrosarcoma	p53 mutations	[24]
		Chromosomal instability + TERT and c-myc expression	[16]
	Undiff. soft tissue sarcomas	Aneuploidy + chromosomal translocations	[17]
Short term culture	Soft tissue sarcomas	Aneuploidy	[18]

Mouse MCSs are reported to spontaneously undergo changes in morphology, proliferation rate, migration ability, cell surface marker profile, genomic constitution and most importantly tumorigenicity after long term *in vitro* culture [4,9,20,21]. Meanwhile, one study has also revealed that mMSCs could transform even after short term *in vitro* culture. Injection of passage 3 mMSCs into mice resulted in formation of tumours comparable with soft tissue sarcomas [19]. Transformed mouse MSCs always show a higher proliferation rate than the native cells [3,4]. These transformed cells exhibit tumorigenicity, as shown by anchorage-independent growth assay and xeno-transplantation in mice and zebrafish, while this is not observed with low passage mouse MSCs before their transformation [3,4,6,22]. Interestingly, the readiness of *in vitro* tumorigenic transformation seems to be a unique property of mouse MSCs since it is absent in most other mouse stem cells, including hematopoietic stem cells and embryonic stem cells [23]. This readiness can be probably ascribed to the genetic instability already shown in mouse MSCs very shortly after isolation from bone marrow, although the cytogenetic abnormalities in low passage mouse MSCs are considerably less in number than in transformed mouse MSCs [23]. Interestingly, MSC spontaneous transformation happens much less frequently *in vivo*, as shown by the low incidence of spontaneous sarcomagenesis in mice. This can be possibly explained by the different microenvironment of *in vitro* and *in vivo* conditions for MSCs. Solid research on the role of the *in vivo* niche of BMMSCs in guarding its genomic stability is needed to answer this question more exactly.

### Induced mouse MSC transformation

Transformation of mouse MSCs has been induced by an array of manipulations, including knockout of tumour suppressor genes, overexpression of oncogenes and drug administration to affect signaling pathways. The pathways targeted by these manipulations are mostly involved in cell cycle checkpoint control, cell survival, proliferation and apoptosis (Table 2) [14]. In one study, loss of tumour suppressor *P21* and *Tp53* in mouse adipose derived MSCs (AMSC) induced *in vitro* transformation and *in vivo* so-called fibrosarcoma formation after transplantation [24]. In another study, both *Tp53−/− Rb−/−* and *Tp53*−/− mouse AMSCs were generated through Cre mediated excision of loxP flanked loci. Leiomyosarcoma-like tumours were developed in the *in vivo* tumorigenicity assays of these 2 types of mouse AMSCs [8]. The combination of *Cdkn2a* loss and *C*-*myc* overexpression in mouse BMMSCs gave rise to osteosarcomas accompanied by the loss of adipogenic differentiation capacity in transformed mouse BMMSCs [16]. Besides directly targeting *in vitro* cultured MSCs, several genetically engineered mouse models have been developed to investigate the effects of genes on transformation process. A conditional mouse model with *Tp53* homozygous deletion has been created by crossing *Prx1*-Cre transgenic mice to mice bearing alleles of *Tp53* flanked by loxP. Prx1 is specifically expressed in the early mesenchymal tissues of embryonic limb buds [17]. In these P53-deficient mice many types of sarcomas occurred in the mesenchymal cells of limb buds and osteosarcoma was the most common type. A mouse model with loss of RB generated also through Cre-loxP system was not found to display tumorigenesis. However, loss of RB accelerated tumorigenesis in P53-deficient mice [17]. These induced transformation studies established the importance of the P53 pathway in preventing mouse MSC transformation. Besides, in spontaneous transformation studies of mouse MSCs, defects in *Tp53* or *Cdkn2a* genes were frequently found [18]. P53 and P14, proteins encoded by these two genes, are both important members of P53 pathway, further corroborating the crucial role of P53 pathway in mouse MSC transformation [4,25]. Upregulated oncogenic pathways have also been shown to induce or potentiate mouse MSC transformation. *Fos* is an oncogene encoding a transcription factor downstream of many growth factor pathways. The *Fos* overexpression transgenic mice resulted in the development of bone tumours, with chondrosarcomas as the main type [26]. This is puzzling as the driver mutation in human central chondrosarcoma is IDH1 or IDH2 [27], while in peripheral chondrosarcomas this is not known [27,28], but no indication for involvement of Fos is found [28,29]. The PI3K-AKT pathway is crucially involved in apoptosis and proliferation. In a study a mouse model with homozygous loss of *Pten*, a negative regulator of the PI3K-AKT pathway in smooth muscle lineage cells developed leiomyosarcomas [30,31]. The MAPK pathway is principally responsible for mitosis. Overexpression of *K*-*ras*, a component of the MARK pathway in addition to P53 loss induced sarcoma formation in mice more efficiently than in mice with P53 loss alone [32,33]. These studies underscore the role of different oncogenic pathways to promote mouse MSC transformation.

**Table 2:** A summary of induced transformation studies with mouse cells

**Sarcomas without specific chromosomal translocation**				
**Cell type**	**Inactivated gene(s)**	**Expressed gene(s)/treatment**	**Type of sarcoma**	**Reference**
mASCs	p21 + p53	-	“Fibrosarcoma”	[20]
	p53 or p53 + Rb	-	Leiomyosarcoma	[7]
BM-mMSCs	INK4A/ARF	c-myc	Osteosarcoma	[24]
Osteoblastic lineage	p53 or p53 + Rb	-	Osteosarcoma	[32,33]
Mesenchymal cells of limb buds	p53	-	Osteosarcoma	[23]
	p53 + Rb	-	Undifferentiated sarcoma	[23]
Muscle, uterus	p53 orINK4A/ARF	K-RAS	High-grade sarcoma with myofibroblastic differentiation	[25]
Muscle	p53	K-RAS	Pleomorphic rhabdomyosarcoma	[18]
Smooth muscle lineage	PTEN	-	Leiomyosarcoma	[17]
MSC progenitors	APC	-	Aggressive fibromatosis	[67]
**Sarcomas with specific chromosomal translocation**				
**Cell type**	**Expressed fusion gene**		**Type of sarcoma**	**Reference**
BM-mMSCs	EWS-FLI-1		Ewing sarcoma	[63]
	EWS-FLI-1		Ewing sarcoma	[65]
	FUS-CHOP		Myxoid liposarcoma	[11]
	PAX3/7-FKHR		Alveolar rhabdomyosarcoma	[71]
mASCs	FUS- CHOP		Liposarcoma	[11]
Mesenchymal cells of limb buds	EWS-FLI-1		Ewing sarcoma	[65]
Differentiated muscle cells (MYF6-expressing cells)	PAX3-FKHR		Liposarcoma	[70]

### Human MSC transformation

Human MSCs have not been shown to undergo spontaneous transformation *in vitro*[9,15,43]. There have been few reports on spontaneous human MSC *in vitro* transformation, of which two turned out to be caused by contamination by tumour cell lines and were retracted afterwards [34,35,44]. Meanwhile, there are several studies demonstrating that human MSCs did not go through transformation in spite of long term *in vitro* culturing [12,15]. For the possibility of *in vivo* spontaneous transformation, there have been few cases of osteosarcoma genesis in patients infused with bone marrow MSCs for other diseases [45-47]. The majority studies of human MSC transformation are based on genetic approaches to knock out important tumour suppressor genes and overexpress certain oncogenes Table 3) [14]. In contrast to mouse MSC studies, four of the induced human MSC transformation studies consist of the exogenous expression of *hTERT* in human cells [38,48-50]. This may be attributed to the much shorter telomeres in human MSCs than their mouse counterparts, the much shorter life span of mice than human and the difference in telomere damage signaling pathways between mouse and human [41,50,51]. Consistent with mouse MSC studies, the disruption of cell cycle control machineries, exemplified by P53 and RB pathways are also important for human MSC transformation. For instance, the introduction of *SV40*-*LT*, which perturbs both P53 and RB proteins potently promoted human MSC transformation [38]. Furthermore, the overexpression of some oncogenes has also been shown to contribute to the transformation, such as *H*-*RAS*[5-7]. Although the definite spontaneous transformation capacity of mouse MSCs is not a mimicry of human MSCs, the signaling pathways underlying their tumorigenic transformation show high consistency, including the P53 pathway, RB pathway, PI3K-AKT pathway and MAPK pathway and so on.

**Table 3:** A summary of human BMMSCs transformation without specific chromosomal translocation

**Type of sarcomas**	**Expressed gene(s)/treatment**	**Reference**
Undifferentiated spindle cell sarcoma	hTERT + HPV16 E6/E7 + SV40-ST + H-RAS	[44]
	hTERT + SV40-LT + H-RAS	[13]
	hTERT + H-RAS + BMI-1	[43]
Tumors with smooth muscle and bone properties	hTERT4	[33]
Undifferentiated pleomorphic sarcomas	DKK1 + SV40-LT	[14]

## MSCs as the origin of sarcomas and tumour type specificity

There is substantial evidence supporting a MSC origin of a spectrum of sarcomas, both pleomorphic as well as translocation driven subtypes. In the non-translocation-driven sarcoma types, the correspondence between the differentiation capacity of MSCs and the histological spectrum of different types of sarcomas is reflected (Figure 1). Approaches and methods have also been used to investigate this hypothesis, including differentiation assays, expression profiling and Immunohistochemistry [52-54]. Based on the site of presentation, sarcomas can be categorized into bone tumours and soft tissue tumours. Based on genetic profiles, sarcomas can be categorized into two groups, one with relatively simple genetic alterations, either being associated with point mutations or reciprocal translocations, and the other with extensive genetic changes. Examples of the cytogenetically relatively simple group are alveolar rhabdomyosarcoma, myxoid liposarcoma, Ewing sarcoma and synovial sarcoma. Examples of the other group are leiomyosarcoma, undifferentiated pleomorphic sarcoma and osteosarcoma [55].

**Figure 2:**
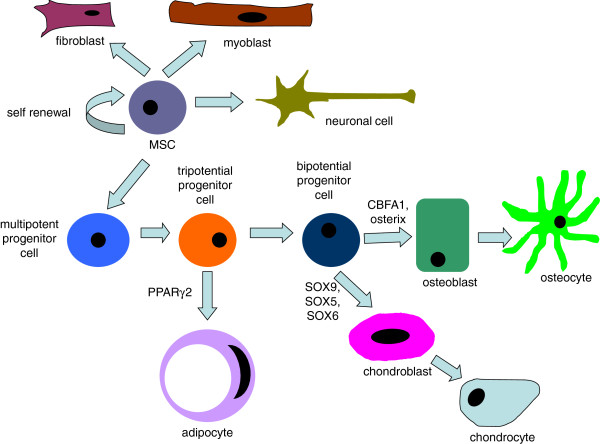
**MSC differentiation scheme.** Under different signaling regulations, MSCs can differentiate into different types of cells. The differentiation process involves sequential signaling regulation and many different stages.

MSC differentiation towards a defined and differentiated cell type is a process with a lot of different signaling pathways and differentiation stages involved (Figure 2). The sarcoma type arising from *in vitro* transformed MSCs after inoculation into mice seems to be dependent on many factors, including the originating tissue of the MSCs, the differentiation commitment status of the targeted cell and also the targeted molecular pathways. In most cases with bone marrow derived mouse MSCs (BMMSC) or osteochondro progenitors osteosarcoma-like tumours were formed. With AMSCs or smooth muscle cell progenitors leiomyosarcomas were mostly formed (Table 4) [8,16,24]. BMMSCs from aged mice tend to spontaneously give rise to so-called fibrosarcomas instead of osteosarcomas as in most spontaneous transformation studies [25]. It must be added that according to the present view fibrosarcomas is a poorly defined histological entity. It is necessary to perform large scale studies to specifically address the relationship between tissue origin, targeted pathways and the sarcoma type generated, which is currently lacking.

**Table 4:** A summary of sarcoma types from different transformation studies in mice

**Sarcoma type**	**Cell of origin**	**Targeted genes**	**Reference**
Osteosarcoma	Mouse BMMSCs	-	[4]
		-	[6]
		*C*-*myc* overexpression and *Ink4a*/*Arf* knockout	[16]
	Mouse osteoblast precursors	*Tp53*, *Rb* double knockout	[56]
	Mouse osteoblasts	*Tp53*knockout	[57]
		*Tp53* and *Rb* double knockout [57]	
		*Tp53* knockout	[17]
Leiomyosarcomas	Mouse AMSCs	*Tp53* knockout	[14]
		*Tp53* knockout	[24]
		*Tp53* and *Rb* double knockout [8]	
	Mouse smooth muscle lineage cells	*Pten* knockout	[30]
“Fibrosarcoma”	Mouse BMMSCs	-	[58]
	Aged mouse BMMSCs	-	[25]
	Mouse AMSCs	*P21* knockout, *Tp53* heterozygous knockout	[24]
Pleomorphic rhabdomyosarcoma	Mouse skeletal muscle cells	*K*-*ras* overexpression and *Tp53* knockout	[32]
		*K*- *ras* overexpression and *Tp53* heterozygous knockout [32]	

### Bone sarcomas

#### Ewing sarcoma

Ewing sarcoma arises predominantly in bone but in soft tissues as well. It is a type of a poorly differentiated tumour known to be associated with a the expression of *EWSR1*–*ETS* fusions or rarely other chimeras [59-62]. The exogenous expression of the fusion gene *EWS*-*FLI1* alone in mouse MSCs has been shown to transform these cells, demonstrated by *in vitro* immortalization and *in vivo* sarcomatous tumour formation after inoculation in immunocompetent mice [63]. In another study a secondary genetic alteration was needed for the induced transformation of mouse MSCs [64]. Similar manipulations have been also applied on human MSCs. Human MSCs with exogenous *EWS*-*FLI1* expression transformed and these transformed cells expressed neuroectodermal markers [65]. Moreover, the knockdown of *EWS*-*FLI1* expression in Ewing sarcoma cell lines restored the *in vitro* trilineage differentiation ability of the cells [52]. In a transgenic mouse model, by expressing *EWS*-*FLI1* gene specifically in the mesoderm-originated tissues in limbs and simultaneous *Tp53* knockout, sarcomas with similar characteristics as Ewing sarcoma occurred while with only *Tp53* knockout the primary sarcoma type was osteosarcoma [66]. In brief, Ewing sarcoma, originally considered as tumours arising from the neuroectodermal lineage and not considered of mesenchymal origin could be experimentally derived directly from MSCs, but only upon introducing the typical translocation. This strongly supports an MSC origin of Ewing sarcoma [67].

#### Osteosarcoma

Osteosarcoma is the most common primary malignant bone tumour among children. It is characterized by the production of osteoid and extensive cytogenetic instability [36]. Different studies have supported the MSC origin of osteosarcoma [4,16]. Both spontaneous and induced MSC models for osteosarcoma have been discussed above. Osteosarcomas mainly arise in the metaphyses of long bones and the peak incidence is in the second decade of human life, correlating with the rapid bone growth during puberty, a process in which MSCs are crucially involved [37]. In both human osteosarcoma cells and transformed MSCs, frequent aberrations in genes encoding components of P53 pathway have been identified [4,39]. In *Tp53* knockout mice many types of sarcomas developed and osteosarcoma was the main type [17].

#### Chondrosarcoma

A study compared the gene expression profiles of chondrosarcomas of different differentiation degree [53]. Less differentiated chondrosarcomas were shown to have more similarity with MSCs of pre-chondrogenic stages and more differentiated chondrosarcomas share more similarity with fully differentiated chondrocytes. This suggests that chondrosarcoma progression probably parallels deregulated chondrocytes differentiation process of MSCs [40,53].

### Soft tissue sarcomas

#### Synovial sarcoma

In synovial sarcoma, exogenous expression of SYT-SSX2 fusion gene in the skeletal-muscle-specific Myf5 expressing lineage induced the formation of synovial sarcomas *in vivo*. Remarkably, when this fusion gene was introduced into cells more differentiated than myoblasts synovial sarcoma did not occur [68]. This fact emphasizes the important role of cell status in the genesis of specific type of sarcomas. On the other hand, fusion gene silencing in primary synovial sarcoma cells restored both the trilineage differentiation capacity and the MSC marker expression, strongly suggesting cells of MSC lineage as the origin of synovial sarcoma [69]. This may be explained by the fact that although considered as muscle specific Myf5 can also be expressed in some MSCs during development.

#### Other soft tissue sarcomas

Similar results as described above were seen in a mouse model of liposarcoma, where FUS -*CHOP* was able to induce liposarcoma genesis in MSCs, whereas no liposarcoma was formed when *FUS*-*CHOP* gene was manipulated to be only expressed in differentiated, aP2-expressing adipocytes. This study again underscores the exact cell status as a crucial factor in sarcomagenesis [42,70]. However other studies show that there is considerate plasticity in the different lineages since rhadomyosarcoma, an aggressive skeletal muscle tumour can be generated from adipocytes by activation of Sonic Hedgehog signaling [71]. A third soft tissue sarcoma model is that of clear cell sarcoma, characterized by melanoma-like features and an EWSR1/ATF1 translocation. Conditional expression of the human EWSR1/ATF1 fusion gene in mouse gives rise to tumorigenesis with extreme brief latency. The most stem-like MSCs give rise to fully melanoma-like lesions, whereas more differentiated cells result in a less clear cell sarcoma phenotype [72].

## Discussion

Until now there have not been many studies addressing the effect of MSCs of different tissues and different ways of preparation on the role of MSCs as a model for sarcoma genesis. The conspicuous difference between mouse and human MSCs in spontaneous transformation can be possibly explained by many factors. In human cells, the telomeric DNA is often 5–10 kb long and mouse cells have a telomeric DNA length of 30–40 kb [41,51]. The longer telomeres in mouse MSCs allow cells to proliferate many generations before reaching reaching the telomere length limit, giving higher chance for cell to acquire aberrations [41,51]. Since mice have a shorter life span than humans, the genome maintenance in mouse cells is also less stringent than in human cells [73].

Niche is one of the most important factors in the determination of stem cell characteristics. The function of niche in stem cell differentiation and pluripotency maintenance is well known. There has also been research showing that the low oxygen tension is important for multipotency maintenance of MSCs, while normal oxygen level will induce differentiation [74]. Besides, niche has also been indicated to be involved in tumorigenesis [75]. This suggests the important role of niche in genomic instability and therefore tumourigenetic ability. One special feature of the bone marrow niche is the partnership of MSCs and haematopoietic stem cells, which deserves further exploration [76,77].

## Future considerations

The numerous but well documented studies on MSCs giving rise to sarcomas in experimental set-up provide excellent models to study this devastating malignancy in a systematic and controlled way. This offers opportunities for preclinical testing of experimental therapies, thereby providing convincing data that may facilitate application in actual clinical trials despite small patient cohorts.

## Conclusions

Although mouse MSCs have exhibited definite readiness to transform in vitro, human MSCs do not go through transformation in ex vivo expansion and need additional manipulation before progression into sarcomas. Therefore, although there are few cases of osteosarcoma genesis in patients infused with bone marrow MSCs [45-47], it is considered generally safe to use human MSCs in clinic.

## Abbreviations

AKT: v-akt murine thymoma viral oncogene homolog; AMSC: Adipose tissue derived MSC; APC: Adenomatous polyposis coli; BMI-1: B lymphoma Mo-MLV insertion region 1 homolog; BMMSC: Bone marrow derived MSC; C-myc: v-myc myelocytomatosis viral oncogene; Cdkn2a: Cyclin-dependent kinase inhibitor A; CHOP: C/EBP homologous protein; DKK1: dickkopf 1 homolog; EWS: Ewing Sarcoma; ETS: E-twenty six; FKHR: Forkhead in rhabdomyosarcoma; FLI1: Friend leukemia integration 1; Fos: FBJ murine osteosarcoma viral oncogene; FUS: Fused in sarcoma; H-RAS: v-Ha-ras Harvey rat sarcoma viral oncogene homolog; HLA-DR: Human leukocyte antigen-DR chain; HPV: Human papillomavirus; hTERT: Human telomerase reverse transcriptase; IDH: Isocitrate dehydrogenase; INK4A/ARF: Inhibitor of CDK4 A/Alternative Reading Frame; K-ras: v-Ki-ras2 Kirsten rat sarcoma viral oncogene homolog; MAPK: Mitogen-activated protein kinase; MSC: Mesenchymal Stem Cell; MYF6: Myogenic factor 6; PAX3/7: Paired box 3/7; PI3K: Phosphatidylinositol-3-kinase; Prx1: Peroxiredoxin 1; Pten: Phosphatase and tensin homolog; Rb: Retinoblastoma; SSX2: Synovial sarcoma translocation 2; SV40-LT: Simian virus 40-large antigen; SYT: Synaptotagmin; TERT: Telomerase reverse transcriptase.

## Competing interests

The authors declare that they have no competing interests.

## Authors’ contributions

Wei Xiao drafted the manuscript. Alexander B. Mohseny, Anne-Marie Cleton-Jansen and Pancras C. W. Hogendoorn provided comments and suggestions. All authors read and approved the final manuscript.
